# Paraffin embedding of the whole human cerebral hemisphere to assess arterial distribution territories

**DOI:** 10.17179/excli2023-6601

**Published:** 2024-04-29

**Authors:** Mykyta Smirnov, Frédéric Andersson, Laurent Barantin, Igor Lima Maldonado, Christophe Destrieux

**Affiliations:** 1 Université de Tours, INSERM, Imaging Brain & Neuropsychiatry iBraiN U1253, 37032, Tours, France; 2 BAOBAB/NeuroSpin, Université Paris-Saclay, CNRS, CEA, Gif-sur-Yvette, France; 3 CHRU de Tours, Le Centre Hospitalier Régional Universitaire de Tours, Tours, France

**Keywords:** tissue processing, whole hemisphere, volume reconstruction, paraffin embedding, brain vascularization, arterial distribution

## Abstract

Commonly used to decode the human brain's structural complexity, *ex vivo* dissection focuses on a given structure or region but cannot depict the whole brain organization (for example, its arterial distribution territories). Where dissection reaches its limit, the combination of tissue sectioning and 3D reconstruction may provide a volume for the assessment of structures from any view angle, following them dynamically to understand their spatial relationships. However, to produce sections, standard histological tissue processing protocols for paraffin embedding cannot be applied to a cerebral hemisphere as the latter is extensively larger than the conventional specimens. This paper presents a protocol for paraffin embedding of the whole human cerebral hemisphere and a method to reconstruct 3D volumes from serially sectioned and photographed paraffin blocks containing embedded hemispheres. Seven *ex vivo* whole human cerebral hemispheres were included, two were serially sectioned. Main cerebral arteries were injected with colored media to label arterial territories. A detailed description of every step, from tissue processing to image acquisition of cut blockfaces and volume reconstruction, is provided. Tissue processing and section cutting were reproducible, and the former provided complete and homogeneous paraffin wax impregnation. 3D visualization of the reconstructed whole human cerebral hemisphere successfully showed the distribution territories of the main cerebral arteries. In addition, we discuss the challenges we faced and overcame while developing the presented method and highlight its originality.

## Introduction

The brain complexity can be decoded *ex vivo* by dissection or section cutting. Although dissection usually focuses on a given structure or group of structures which it describes in detail, it cannot depict the whole brain organization. And, when dissection comes to studying an objective distributed over the whole brain, for instance its vasculature, it reaches the limit. The combination of tissue sectioning and 3D reconstruction provides a volume for assessment of structures from any view angle, following them dynamically to understand their spatial relationships. Additionally, depending on the studied structure, volumes can be observed at a variable scale, from macroscopic to microscopic.

However, to produce sections, standard histological tissue processing protocols for paraffin embedding cannot be applied to a whole human cerebral hemisphere (WHCH) as the volume of the latter is hundreds of times larger than of the usual specimens - around 1-2 cm^3^ (Shan et al., 2005[26]). While some cases of WHCH embedding were reported (Amunts et al., 2013[2]; Huron Digital Pathology, 2017/ 2020[14]), the authors did not provide a detailed methodology as was described for the smaller macaque brain (Zhanmu et al., 2020[32]).

Such a voluminous approach is especially important for studying the deep blood supply and complex architecture of the brain vasculature. These may be labeled with our recently developed method (Smirnov et al., 2023[28]) to inject the main cerebral arteries with colored gelatin in conditions mimicking physiological perfusion (continuous, at a physiological pressure level, and simultaneous).

This paper presents a detailed method for 1) paraffin embedding of WHCH and 2) its volume reconstruction after section cutting and blockface photography. To demonstrate the method's application for assessing arterial distribution territories, the main cerebral arteries were injected with colored media (Smirnov et al., 2023[28]).

## Materials and Methods

Seven human cerebral hemispheres (4 male brains, mean age: 88.4 ± 10.7 years, min.: 78, max.: 98) were obtained from the University of Tours body donation program. Before death, participants provided written testaments of their willingness to become body donors and consent to using their bodies for educational or research purposes. The study was approved by the board of the University of Tours body donation program, and the authors followed local and international ethical guidelines and laws that pertain to the use of human cadaveric donors in anatomical research.

### Brain extraction and injection

Not later than 48 hours *post mortem,* brains were extracted. Using an electrical surgical craniotome equipped with a fluted spiral saw (ANSPACH™ EMAX™ 2 PLUS Electric System, New Brunswick, NJ, USA) ensured the preservation of the brain surface during the procedure. The anterior and middle cerebral and basilar arteries were selectively catheterized and flushed with isotonic saline solution until no blood visually remained in the vessels. Then, we injected 150 ml of 4 % formaldehyde (Formaldehyde 4 % w/v buffered at pH 6.9 RS, Carlo Erba Reagents, Cornaredo, Italy) in each hemisphere for primary fixation (Latini et al., 2015[16]).

Subsequently, we injected arteries with colored gelatin, as was previously described (Smirnov et al., 2023[28]). Briefly, gelatin from porcine skin (gel strength 300 Bloom, Type A, Sigma-Aldrich, St. Louis, MI, USA) was mixed with colored dry pigments: red, blue, and green (respectively, *SP rouge 55* azo pigment, *SP bleu 7* copper-phthalocyanine pigment, and *SP vert 8* halogenated copper-phthalocyanine pigment; Color Rare, Villenave d'Ornon, France). Then, the mixtures were simultaneously injected at an identical and constant pressure (140-160 mmHg) using an injection/heating apparatus. 

Subsequent immersion of the brains in 4 % buffered formaldehyde solution for two months at ambient temperature provided complete specimen fixation. To limit distortion, we hang the encephalon in fixative by a thread running around the basilar artery keeping it away from the container walls.

### WHCH paraffin embedding

**Figure 1:**
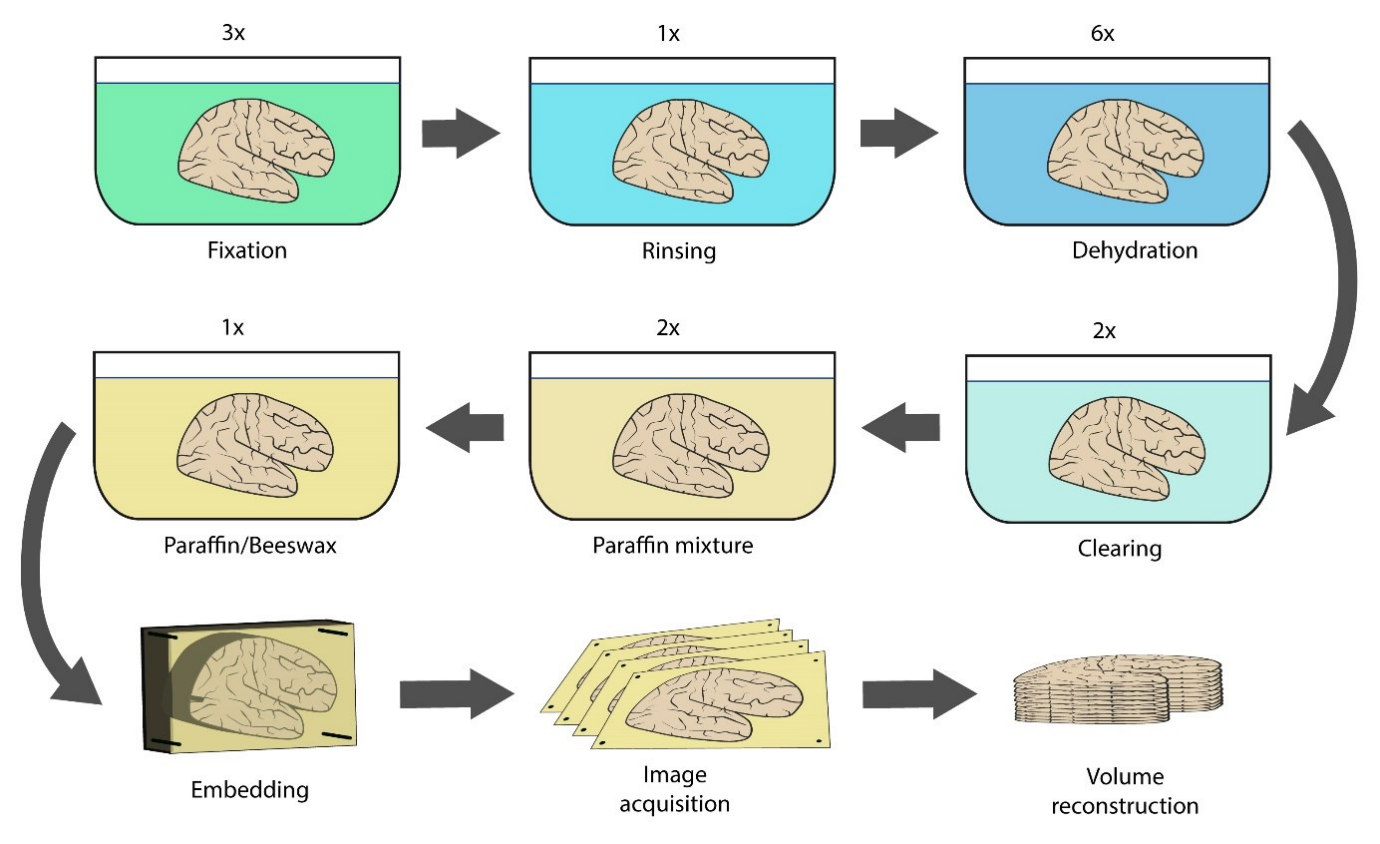
Schematic representation of method's workflow: 3 baths of 4% formaldehyde >> 1 bath of tap water >> 6 baths of ascending ethanol concentrations >> 2 baths of xylene (transition bath ommited) >> 2 baths of paraffin mixtures >> 1 bath of paraffin/beeswax mixture >> paraffin block solidification >> cutting and image acquisition >> image preprocessing and volume reconstruction

For WHCH paraffin embedding, we modified a standard paraffin processing method (Dey, 2018[7]). The baths, their composition, and duration are detailed in Table 1(Tab. 1), and the general workflow - in Figure 1(Fig. 1). All the baths were larger, and specimens were introduced for a longer time as compared to conventional schedules. Full protocol for WHCH paraffin embedding and blockface data acquisition is available in Supplementary information. 

First, the hemispheres were immersed in tap water for two days to remove excess formaldehyde. Then, they were dehydrated in six successive baths of ascending ethanol concentrations (Ethanol absolute anhydrous RE - Pure, Carlo Erba Reagents, Cornaredo, Italy), put in a transitional bath (absolute ethanol and xylene, 50 %/50 %), and finally cleared in two successive xylene baths (Xylene, mix of isomers RE - Pure, Carlo Erba Reagents, Cornaredo, Italy). The dehydration baths were continuously gently agitated using an orbital shaker (VWR Standard Orbital Shaker, Model 1000, VWR International, Radnor, PA, USA). 

For paraffin infiltration, we used two successive baths of melted paraffin wax mixture (Paraffin 42-44 MP, Merck, Kenilworth, NJ, USA; Histoplast 57-58 MP, Thermo Scientific, Waltham, MA, USA; proportions 50 %/ 50 %). They were followed by one bath of melted paraffin/beeswax mixture (Paraffin 42-44 MP; Histoplast 57-58 MP; Beeswax, white, technical, VWR Chemicals, Radnor, PA, USA; proportions 60 %/25 %/15 %). We used a laboratory oven to maintain paraffin at an optimal temperature (Table 1(Tab. 1)).

We applied a -45000 Pa pressure (Hallas, 1975[12]) to the last two dehydration baths (absolute ethanol) and final paraffin/beeswax bath: baths were placed under a glass dome connected to the lab's vacuum system through a manometer. For the last paraffin/beeswax bath, we put this glass dome in the oven.

Specimens were embedded in the same final paraffin/beeswax mixture in heat-resistant plastic molds. They were placed in a refrigerator (at 4-6 °C) to speed up solidification. The paraffin blocks remained at the ambient temperature for at least two weeks before being trimmed and mounted on the specimen holder. Then, we drilled four holes perpendicular to the holder at the corners of the paraffin block. We filled them with black-colored paraffin to obtain high-contrast fiducial markers for future image processing and block reconstruction.

### Section cutting / Data acquisition

Two paraffin blocks were mounted on a specimen holder and cut on a large-scale heavy-duty sliding microtome (Leica SM2500, Leica Biosystems, Nussloch, Germany) every 20 μm. This device comprises a sliding holder that allows translation of the specimen in the horizontal plane and a knife holder that vertically sets the slice thickness. After every cut, the knife moves down for this distance, and the holder moves the specimen towards it. We used 16 cm type C knives (Leica Biosystems, Nussloch, Germany) set to a 5° clearance angle from the specimen surface. 

**Figure 2:**
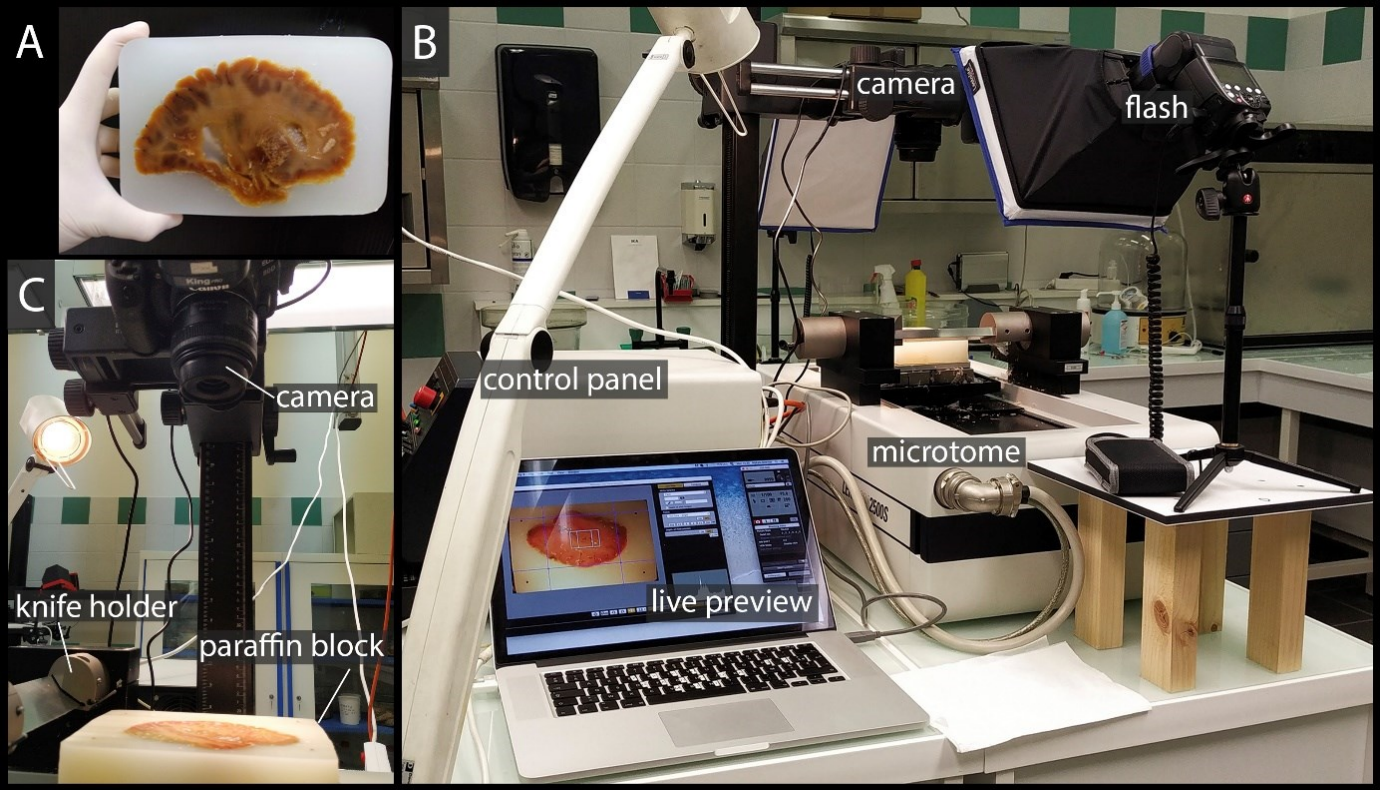
Data acquisition set-up. A: Blockface of the paraffin block with embedded whole human cerebral hemisphere being held in a hand. This specimen was not injected with colored gelatin and was used during the methodology development. One may also notice the absence of fiducial markers in the corners of the block. B: Section cutting and photography set-up. The camera is operated remotely from the computer, enabling the live preview of the photograph. On the sides of the microtome two flashes are equipped with diffusers to provide homogeneous lightning on the blockface. C: Digital camera is placed perpendicularly above the paraffin block in the fixed position. Knife holder is on the left from the specimen.

A digital single-lens reflex camera (Canon 80D, Canon, Tokyo, Japan) equipped with a macro lens (Canon EF-S 35 mm f/2.8 Macro IS STM, Canon, Tokyo, Japan) was perpendicularly fixed above the microtome. Due to the size of the structure of interest and to limit the size of the data, we photographed the blockface of each tenth cut (inter-photographs space: 0.2 mm) at the end of a cutting cycle (default position). The camera and microtome were strictly horizontal to limit angulation-induced distortion (Figure 2C(Fig. 2)). Lighting was carefully controlled to get homogenous exposure throughout the images (2 flashes equipped with diffusers pointed on the blockface with a 30° angle to avoid reflections). The camera was controlled from a MacBook Pro (Apple, Cupertino, CA, USA) running the EOS Utility 3 (Canon, Tokyo, Japan) software (Figure 2B(Fig. 2)). We set the following camera parameters: aperture - 5.0, ISO - 100, shutter speed - 1/60, white balance - K5500, picture style - fine detail, HDR (high dynamic range) mode - disabled, WB (white balance) shift - "0,0", image quality - L (6000 x 4000 px, file extension - .jpg), metering mode evaluative, drive mode - single shooting. With a field-of-view of 150 x 100 mm and image resolution equal to 6000 x 4000 px, the pixel size resulted in 0.025 x 0.025 mm.

### Volume reconstruction

Blockface images were coregistered using in-house MATLAB scripts. First, the center of each fiducial marker was identified ​​using the algorithm based on a circular Hough transform (Duda and Hart, 1972[8]; Hough, 1962[13]; Kimme et al., 1975[15]). Then, according to the inter-markers distance, we computed the magnification factor for each blockface image to correct the scale factor due to changes in the camera-specimen distance induced by the cutting process. Finally, based on the positions of markers in each blockface image, we estimated geometric transformations (rotations and translations) onto the first image (registration target).

We used a custom program written in Python (version 3.10.2, 64-bit) for the image preprocessing and volume reconstruction. Program leaned on PySimpleGUI library (“PySimpleGUI,” 2022[21]), version 4.60.4, to enable a user-friendly graphical interface. 

#### Preprocessing pipeline:

in-plane resolution of blockfaces was decreased to 0.2 x 0.2 mm using a Lanczos resampling (Pillow library (Clark, 2015[4]), version 9.0.1, Image.resize() method);

white balance of every blockface was adjusted according to patches of 25 x 25 px located in the same area containing only paraffin (ground truth algorithm (Manansala, 2021[19]) treated patch as "true" white); 

colors were enhanced (Pillow library (Clark, 2015[4]), version 9.0.1, color.enhance() method);

image brightness was adjusted by conversion of color model to HSV, application of the color transfer algorithm (Rosebrock, 2014[24]) to V-channel, and restoring RGB color model.

Finally, volume was rebuilt with 0.2 mm isotropic voxel size from the pile of preprocessed .png images and saved as NIfTI file (SimpleITK library (Yaniv et al., 2018[31]), version 2.1.0, ImageSeriesReader() class).

## Results

Specimens' bathes remained uncolored, and the colored gelatin remained in the arteries at the end of the process, showing that the mixture resisted the entire process (heating, dehydration, solvents, paraffin). On native slices and reconstructed volumes, one can easily define distribution territories of major cerebral arteries marked by the injected colored media, even with the naked eye.

The cut sections remained intact and continuous, meaning that the proposed tissue processing schedule provided complete and homogeneous paraffin wax impregnation of the WHCH. Dark-colored landmarks, having good contrast with the surrounding paraffin and being present in every corner of each 2D image, enabled the realignment of all images in a stack. Depending on the hemisphere's size, 240-260 blockfaces were photographed, resulting in 1.4-1.7 GB of data. Obtained images were sharp, and the contours of various brain structures could be well defined. 

**Figure 3:**
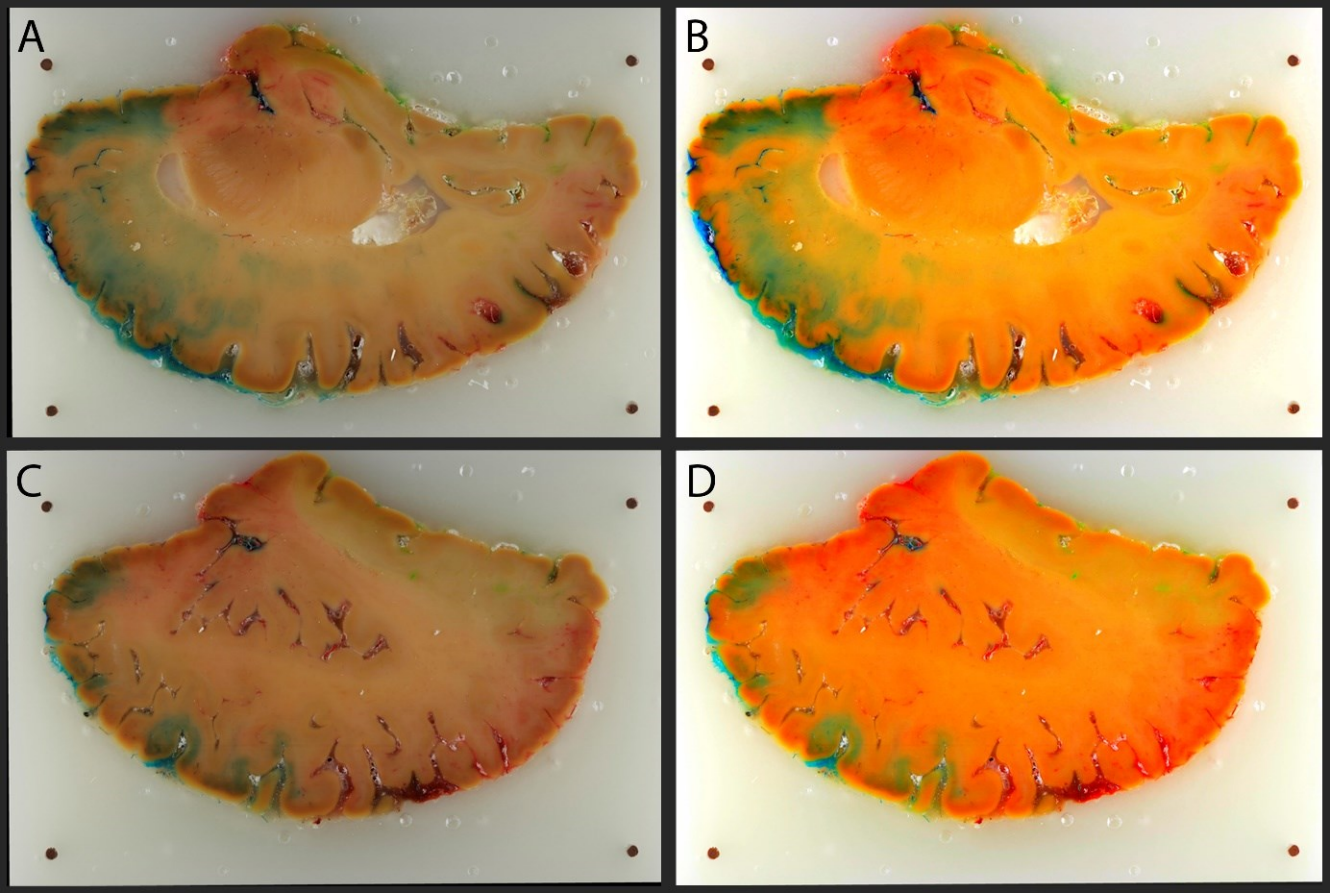
Blockface comparison before and after image pre-processing. A: Section 90, aligned and downscaled. B: Section 90, image pre-processing pipeline applied. C: Section 143, aligned and downscaled. D: Section 143, image pre-processing pipeline applied

Image preprocessing visually ameliorated exposure difference among photographs and enhanced colors making them brighter and easier to distinguish on computer screen (Figure 3(Fig. 3)). 3D RGB volume reconstructed from downscaled images required around 300 MB of storage. The smaller size of the volume enabled its visualization and manipulation, not requiring top-tier computers.

**Figure 4:**
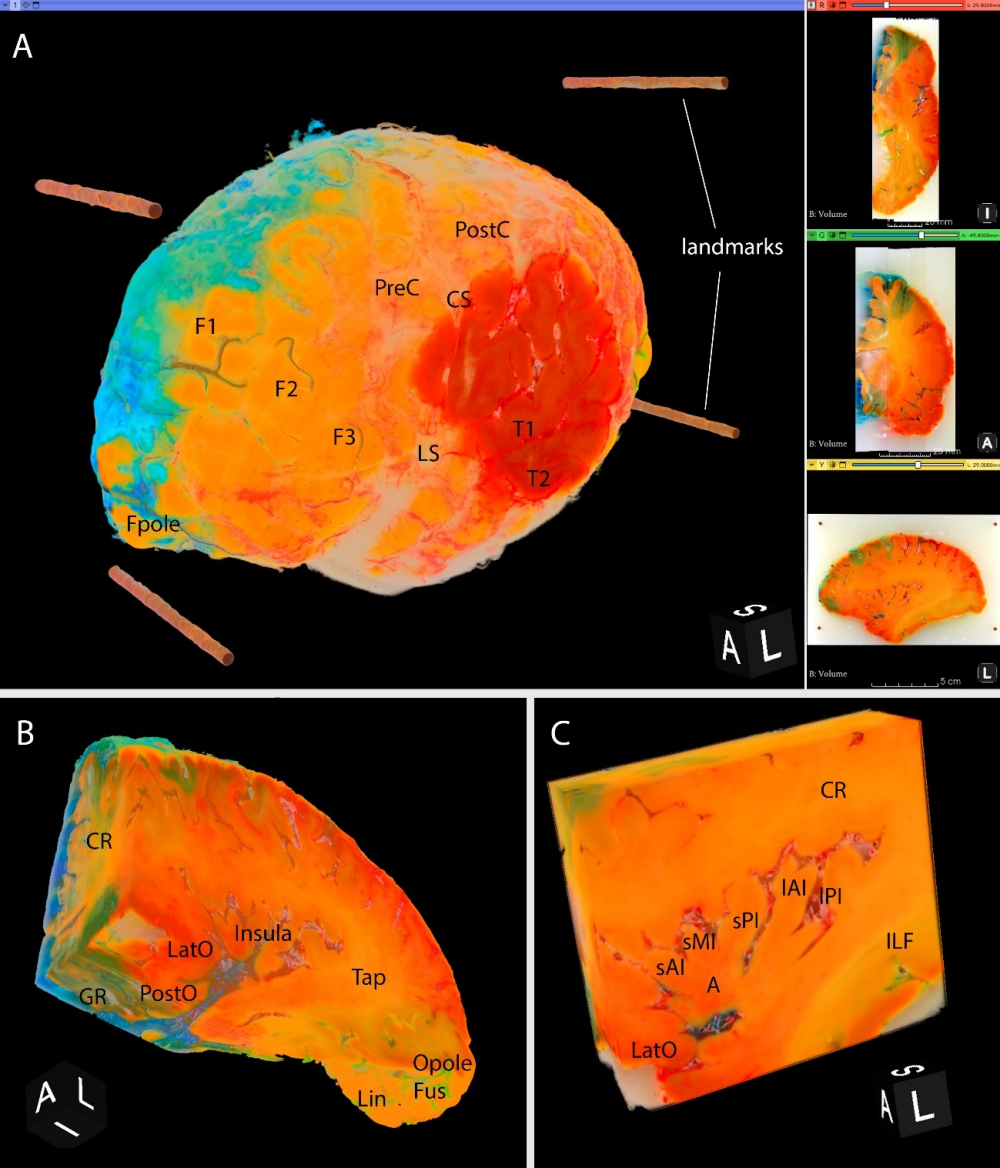
Volume reconstruction in 3D Slicer. Left human cerebral hemisphere injected with blue, red, and green colored gelatin into the anterior, middle, and posterior cerebral arteries, respectively. Cube on the bottom of each image shows specimen orientation. A: Full rendered 3D RGB volume with three 2D views on the right side (horizontal, coronal, and axial from top to bottom). By thresholding almost all surrounding paraffin was omitted. Reconstructed fiducial markers form pillars. Not only the arterial distribution territories of the cortex can be distinguished, but also sulco-gyral pattern and selected vessels. The accumulation of blue colored gelatin between the meninges of the frontal lobe is observed. B: Trimmed block. Coronal view uncovers vascular input to the frontal part of the corona radiata and surrounding structures. Axial view transverses the orbitofrontal cortex. Sagittal view complements visualization of lateral part of the corona radiata and adds the information on the tapetum and the occipital lobe. C: Trimmed block with the focus on insular gyri and sulci in a sagittal view. A = apex of insula, CS = central sulcus, CR = corona radiata, Fpole = frontal pole, F1 = superior frontal gyrus, F2 = middle frontal gyrus, F3 = inferior frontal gyrus, Fus = fusiform gyrus, GR = gyrus rectus, ILF = inferior longitudinal fasciculus, LatO = lateral orbital gyrus, Lin = lingual gyrus, lAI = long anterior insular gyrus, lPI = long posterior insular gyrus, Opole = occipital pole, PreC = precentral gyrus, PostC = postcentral gyrus, PostO = posterior orbital gyrus, sAI = short anterior insular gyrus, sMI = short middle insular gyrus, sPI = short posterior insular gyrus, T1 = superior temporal gyrus, T2 = middle temporal gyrus, Tap = tapetum

3D Slicer (Fedorov et al., 2012[11]) rendered RGB volume showing all three channels and not only one in grayscale (Figure 4(Fig. 4)), the latter being the common limitation of volume visualization software. Preservation of RGB color space during rendering enabled the arterial distribution territories to be observed on the surface and followed interactively by cropping. 

In the specimen presented (Figure 3(Fig. 3), Figure 4(Fig. 4)), blue-colored areas represent distribution territory of the anterior cerebral artery (ACA), red-colored - the middle cerebral artery (MCA), and green-colored - the posterior cerebral artery (PCA). On the surface the territories followed the broadly described and generally accepted pattern (Smirnov et al., 2021[27]). The ACA supplied the middle surface of the hemisphere, except the occipital region, extending to 1-2 cm to the dorsal lateral surface and in the orbitofrontal region of the frontal lobe. The MCA supplied the lateral surface, thus covering the largest territory, and the PCA was limited to ventral regions of the occipital and temporal lobes.

The deep distribution appeared to be more complex than simple extension of superficial territories: borders were mearding and not straight lines as was speculated before (Smirnov et al., 2021[27]), thus the actual deep arterial input was not always obvious (Figure 4(Fig. 4)). For example, deep regions in the projection of the middle and inferior temporal gyri were heavily supplied by the PCA. Interestingly, the inferior longitudinal fasciculus was solely vascularized by the PCA, with a distinct border with the MCA territory along the white matter fibers (Figure 3C, 3D(Fig. 3); Figure 4C(Fig. 4)). Another peculiar observation regarding the PCA; while the subiculum of the hippocampus was supplied solely by the PCA, the hippocampus proper received double supply: CA1 from the PCA and the rest from the MCA (Figure 4B(Fig. 4)). Some regions were still predictable; for instance, the insular region and the underlying internal capsule were vascularised by the MCA (Figure 3B(Fig. 3); Figure 4C(Fig. 4)) branches.

## Discussion

Though large specimen embedding is known (Clarke et al., 2007[5]; Sun et al., 2009[30]; Zhanmu et al., 2020[32]), only partial information (Amunts et al., 2013[2]; Huron Digital Pathology, 2017/2020[14]) can be found in the literature without any precise protocol for the WHCH. One of the objectives of the present paper was to propose a detailed and reproducible embedding method for large specimens.

Paraffin processing aims to impregnate and fully embed the sample with paraffin. It results in a block providing the same cutting resistance in pure paraffin and specimen areas. Considering the specimen volume, automated processing machines were excluded, and the entire routine had to be manual. The final processing schedule, providing better results in the shortest time, was 63 days (after the fixation was completed). If specimens spent less time in baths, they were not fully paraffin-impregnated; the resulting blocks couldn't provide the same resistance throughout the cutting surface. Various methods were proposed in the literature to shorten tissue processing time, such as agitation, heating, microwaves, and using collection devices, handling samples automatically (Lerch et al., 2020[17]; Mishra et al., 2021[20]; Schichnes et al., 1998[25]). To limit tissue alterations (for instance, by microwave) and for safety purposes (toxicity of xylene), we only utilized agitation (Dey, 2018[7]) during the dehydration. On the other hand, longer processing times resulted in specimens being brittle. We added soft and pliable beeswax to the paraffin to make the block texture more prone to cutting (Steedman, 1960[29]).

The formation of bubbles during paraffin solidification is a usual problem, especially for large-volume specimens (Hallas, 1975[12]; Zhanmu et al., 2020[32]). Air bubbles have a dramatically lower density than the surrounding paraffin and may influence the cutting process. In automated processing machines, this issue may be solved by applying a negative pressure to extract dissolved gases from the liquids and specimens (Steedman, 1960[29]). To adapt this approach to the specimens' size, we used a glass dome in which we applied a negative pressure of -45000 Pa during the final dehydration (absolute ethanol) and paraffin/beeswax baths. Once fully impregnated, the specimen surrounded by fused paraffin was stored in a mold at 4-6 °C to limit bubbles formation further.

During large block solidification at the ambient temperature, paraffin's high heat capacity keeps the core of the block melted for a significant time, even when the outer parts are set (Steedman, 1960[29]). When the core's temperature lowers, wax condenses, thus creating negative pressure within the block, which pulls out residual gases. This phenomenon was limited by rapidly cooling the block (the mold was placed in the refrigerator, at 4-6 °C) (Al-Sabaawy et al., 2021[1]). Lower temperatures are nevertheless not advisable, as paraffin may crack.

Another argument for accelerating the paraffin solidification within reasonable limits is the modification of the block's microstructure. In the solid state, paraffin is crystalline, and wax composition (length of carbohydrate chains) and solidification time influence the crystal size: the faster solidification, the smaller crystalline structure, the better cutting experience (Brittig et al., 2004[3]; Steedman, 1960[29]). While the wax composition is predefined by the manufacturer, the time can be manipulated by accelerating the block's cooling, improving its capability of being cut. After initial solidification, the paraffin's crystalline structure continues to change until the paraffin wax sets completely (Steedman, 1960[29]), therefore, the blocks were left to rest for at least 3-4 weeks at the ambient temperature before proceeding to the next step.

No knife's clearance angle is recommended and suitable for all microtome setups. It greatly depends on various factors, such as the knife, its bevel, the microtome, the cut specimen, and the cutting speed. The knife's clearance angle in our setup was 5°.

The various fixation and paraffin embedding steps induce a significant inhomogeneous shrinkage of the specimens. The volume of a mouse brain included in paraffin was recently reported to be 40 % of its value after formalin fixation (Rodgers et al., 2021[23]), with variable shrinkage for the hippocampus and anterior commissure. A smaller shrinkage was also described for the human brainstem, possibly because of different water content (Quester and Schroder, 1997[22]). Using a cryomicrotome (Cocco et al., 2003[6]) is an option to limit specimen shape distortions and the time to process sections. It has other constraints, from the disponibility of a large refrigerated box containing both the specimen and the microtome to managing the condensation on camera lenses. 

To improve the volume reconstruction from serial images and limit thin sections distortion induced by the cutting process, we only considered the blockface whose geometry was preserved despite iterative cuts. We discarded the sections since the microscopy was out of scope and was already reported as feasible for large paraffin-embedded tissues in the macaque (Zhanmu et al., 2020[32]) and human (Amunts et al., 2013[2]) brain. A proper block reconstruction from blockface images is important by itself; it provides information at an intermediate scale, between micro and macroscopic, which is precise enough to study vascular distribution on injected specimens. It is also usually used as an intermediate step to register histological sections onto blockface images, which are less prone to distortion and artifacts (fold, missing slices, tears, sheared slices) (Amunts et al., 2013[2]). This strategy is similar to the one used by optical coherence tomography, which captures the surface of the blockface at the microscopic scale to limit artifacts related to 3D reconstruction from multiple individual sections (Magnain et al., 2014[18]).

Despite the same parameters set and lighting conditions used during the cutting procedure and image acquisition, we observed slight differences in exposure. This was possibly due to the quantity and nature of reflected light from the blockface surface, which may vary along with the cutting phase, depending on the specimen (darker) / paraffin (lighter) ratio. To compensate for these differences, we made an effort to adjust white balance and brightness among images. Also, colors of the original images visually felt muted, thus, we enhanced them as described in methods to ease their perception. Placement of printed reference patches (natural gray or/and several basic colors) with known color values on the blockface before the photo acquisition may facilitate white balance, exposure, and color corrections. 

The distance between the fixed camera and the blockface increased by 200 μm between two consecutive images, which reduced the specimen size on images. A micrometric automated rail may have been coupled to the camera to compensate for these changes in the camera-specimen distance. Nevertheless, slight camera movements also occurred during the cutting session, which resulted in minimal specimen displacement between consecutive blockface images. We used high-contrast fiducial markers, identifiable as black circles in each image, and a coregistration (scaling, rotation, translation) to simultaneously correct size changes and displacements. 

Another objective of this paper was to demonstrate that WHCH tissue processing was compatible with colored gelatin used to mark the arterial territories. Gelatin is fluid at the temperature used for paraffin inclusion, at risk of massive leakage in successive baths. Our results did not confirm this; the pigment-gelatin mixture used for this study remained within the specimen without any significant contamination of the baths. One can hypothesize that the fixation and dehydration processes modified the gelatin structure and blocked it within the vascular compartment. The mesoscopic (i.e., between micro- and macro-) resolution (0.2 mm) of the obtained volume was compatible with the study of brain vascularization, with a relatively easily manageable data size compared to volumes obtained at a microscopic scale. 

In the 1980s, Duvernoy, performed state-of-art anatomical studies - injections of cerebral vessels with black-colored gelatin with subsequent thick 400 µm slices (Duvernoy, 1983[9]; Duvernoy et al., 1981[10]). It was possible to explore the fine 3D organization of the cortical vessels by changing the focus point of a binocular loop within each slice. It was, however, impossible to reconstruct and navigate within a whole-brain volume. By contrast, the present method provides a coherent isotropic 3D volume in which slices can be reconstructed in various planes. 

Since this paper focuses on the complex methodology itself and presents it in maximum detail to ensure its reproducibility, here we only provided the glimpse of WHCH paraffin embedding ability to assess arterial distribution in previously injected specimens. The description of the comprehensive mapping of arterial distribution territories and automated vessel segmentation are intended for forthcoming publications.

The age of the participants (mean: 88.4 years) may be regarded as a possible limitation of the present study. This resulted from the fact that we obtained specimens from an institutional body donation program, and death followed by donation is rarer in the younger population. Another limitation is that the elders may have modified white matter microstructure and vascular environment, but their age is representative of the population suffering from vascular pathology, such as stroke.

Here we presented a method relatively simple to handle a whole hemisphere without any complex equipment. To the best of the authors' knowledge, this is the first time a detailed protocol for specimen fixation, paraffin embedding, and section cutting of a human whole cerebral hemisphere has been published in detail. Tissue processing did not interfere with the mapping of deep territories with colored gelatine, and arterial distribution remained distinguishable both in sections and three-dimensional reconstructions.

## Declaration

### Author contributions

All authors participated in the method development, as well as in the design and revisions of the manuscript. M.S. handled specimens, developed the essence of the text, managed scripts for image processing and figures, and participated in all phases of manuscript creation. L.B. contributed to specimen preparation and problem solving. F.A. was involved in image processing. C.D. and I.L.M. performed additional literature searches, discussed the results, and edited the manuscript.

### Conflict of interest

The authors report no conflict of interest.

### Data availability

Raw data were generated at Inserm U1253 "Imaging and Brain," Tours, France. Derived data supporting the findings of this study are available from the corresponding author on request.

### Acknowledgments

The authors sincerely thank those who donated their bodies to science so that anatomical research could be performed. Results from such research can potentially increase mankind's overall knowledge that would improve patient care. Therefore, these donors and their families deserve our highest gratitude.

In addition, we would like to thank Gerald Deluermoz and Jerome Machut, technicians in the Anatomy laboratory of the University of Tours, for their assistance during the specimen preparation; Markus Cremer, team leader of laboratory in INM-1 division of *Forschungszentrum Jülich*, for confirming the possibility of WHCH paraffin embedding and consulting; and Daniel Bourry for his insights in photography.

### Funding

This study was funded by *Région Centre-Val de Loire (APR Initiative Académique Fibravasc 2017-119973)*, *La Fondation des 'Gueules Cassées'* (78-2020, 71-2022) and *La Fondation Thérèse et René Planiol*. The funding bodies had no role in the study.

## Supplementary Material

Supplementary information

## Figures and Tables

**Table 1:**
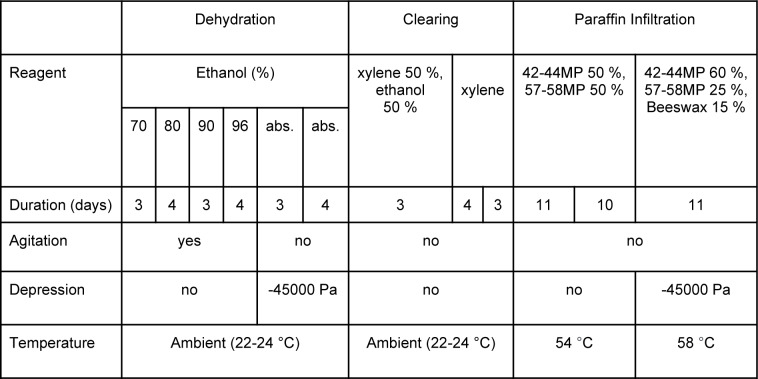
Processing schedule for whole human cerebral hemisphere paraffin embedding
