# Supplementary material for: Paraffin embedding of the whole human cerebral hemisphere to assess arterial distribution territories
Source: EXCLI J. 2024 Apr 29;23:612–23. doi: 10.17179/excli2023-6601 (PMC11180952; doi:10.17179/excli2023-6601)
Supplement: Supplementary information [file EXCLI-23-612-s-001.pdf]

EXCLI Journal 2024;23:612-623 – ISSN 1611-2156
Received: September 20, 2023, accepted: April 18, 2024, published: April 29, 2024

Supplementary information to:

Methods:

PARAFFIN EMBEDDING OF THE WHOLE HUMAN CEREBRAL HEMISPHERE TO ASSESS ARTERIAL DISTRIBUTION TERRITORIES

Mykyta Smirnov,[1,2,*], Frédéric Andersson[1], Laurent Barantin[1],
Igor Lima Maldonado[1], Christophe Destrieux[1,3]

[1] Université de Tours, INSERM, Imaging Brain & Neuropsychiatry iBraiN U1253, 37032, Tours, France
[2] BAOBAB/NeuroSpin, Université Paris-Saclay, CNRS, CEA, Gif-sur-Yvette, France
[3] CHRU de Tours, Le Centre Hospitalier Régional Universitaire de Tours, Tours, France

* **Corresponding author:** Mykyta Smirnov, Université de Tours, INSERM, Imaging Brain & Neuropsychiatry iBraiN U1253, Équipe 3, Faculté de Médecine, Université de Tours, 10 boulevard Tonnellé, 37032 Tours, France; E-mail: mykyta.smirnov@univ-tours.fr

https://dx.doi.org/10.17179/excli2023-6601

This is an Open Access article distributed under the terms of the Creative Commons Attribution License (http://creativecommons.org/licenses/by/4.0/).

PROTOCOL FOR WHOLE HUMAN HEMISPHERE PARAFFIN EMBEDDING AND BLOCKFACE DATA ACQUISITION
(implemented in authors' laboratory)

TISSUE PROCESSING

Important!

- Only trained personnel is allowed to perform the protocol.
- Obligatory use of proper personal protection equipment in accordance with the substances and specimens utilized.
- Used chemicals and materials should be discarded according to the laboratory and local regulations.
- All manipulations with ethanol and xylene should be performed under the fume hood.

EXCLI Journal 2024;23:612-623 – ISSN 1611-2156
Received: September 20, 2023, accepted: April 18, 2024, published: April 29, 2024

- Laboratory ovens for paraffin infiltration should be installed in well-ventilated rooms.
- The container lids should be closed in-between manipulations and as much as possible during the manipulations.

Useful information:

- For steps I, II, and III, use containers with lids appropriate to the chemicals that will be stored in them. Their capacity should be enough to contain at least 6 L of liquid plus the specimen volume and to prevent spilling during agitation.
- For step IV, use containers with lids appropriate to the chemicals that will be stored in them and to the temperatures applied. Their capacity should be enough to contain at least 3 L of liquid paraffin plus the specimen volume.
- For step V, appropriate container/mold should be made fitting the size of the specimen.
- For agitation, orbital shaker (VWR Standard Orbital Shaker, Model 1000, VWR International, Radnor, PA, USA) is used.
- The ambient temperature is considered to be 22-24 °C.
- The temperature in the oven should be maintained at 54 °C for Paraffinmix (see below) and 58 °C for Paraffinwaxmix (see below). Paraffins should be melted in advance to ensure continuity of the tissue processing.
- Respective safety sheets can be found in the laboratory or on the manufacturers'websites.

Chemicals used:

- Ethanol absolute anhydrous RE (Pure, Carlo Erba Reagents, Cornaredo, Italy)
- Xylene, mix of isomers RE (Pure, Carlo Erba Reagents, Cornaredo, Italy)
- Paraffin 42-44 MP (Merck, Kenilworth, NJ, USA)
- Histoplast 57-58 MP (Thermo Scientific, Waltham, MA, USA)
- Beeswax, white (VWR Chemicals, Radnor, PA, USA)

I. Preparation

1. Place the container in the sink and fill it with tap water.
2. Take the previously fixed in formaldehyde specimen from its container and place it in aforementioned container with tap water. Leave it for 24 hours at ambient temperature.
3. Take the specimen from the container, place it aside, change water, and place the specimen back. Leave it for 24 hours at ambient temperature.

II. Dehydration

1. Take the specimen from the water container, gently dry the excess water with gauzes and place it in the first dehydration (70 % ethanol) bath. Place the container on orbital shaker. Leave it for 3 days on minimal speed at ambient temperature.

2. Turn off the orbital shaker. Take the specimen from the first ethanol bath, gently dry the excess ethanol with gauzes and place it in the second dehydration (80 % ethanol) bath. Replace the container on orbital shaker. Leave it for 4 days on minimal speed at ambient temperature.

3. Turn off the orbital shaker. Take the specimen from the second ethanol bath, gently dry its surface from the excess ethanol with gauzes and place it in the third dehydration (90 % ethanol) bath. Replace the container on orbital shaker. Leave it for 3 days on minimal speed at ambient temperature.

4. Turn off the orbital shaker. Take the specimen from the third ethanol bath, gently dry its surface from it from the excess ethanol with gauzes and place it in the fourth dehydration (96 % ethanol) bath. Replace the container on orbital shaker. Leave it for 4 days on minimal speed at ambient temperature. Turn off the orbital shaker. Take the container from the orbital shaker.

5. Take the specimen from the fourth ethanol bath, gently dry its surface from the excess ethanol with gauzes and place it in the fifth dehydration (absolute ethanol) bath. Place the container under the glass dome and using manometer with valve apply -45000 Pa pressure for 3 days at ambient temperature. Carefully normalize pressure. Put the glass dome aside.

6. Take the specimen from the fifth ethanol bath, gently dry its surface from the excess ethanol with gauzes and place it in the sixth dehydration (absolute ethanol) bath. Place the container under the glass dome and using manometer with valve apply -45000 Pa pressure for 4 days at ambient temperature.

7. Then, carefully normalize pressure. Put the glass dome aside.

III. Clearing

1. Take the specimen from the sixth ethanol bath, gently dry its surface from the excess ethanol with gauzes and place it in the ethanol/xylene mixture bath. Leave it for 3 days at ambient temperature.

2. Take the specimen from the ethanol/xylene mixture bath, gently dry its surface from the excess mixture with gauzes and place it in the first pure xylene bath. Leave it for 4 days at ambient temperature.

3. Take the specimen from the first pure xylene bath, gently dry its surface from the excess xylene with gauzes and place it in the second pure xylene bath. Leave it for 3 days at ambient temperature.

IV. Paraffin infiltration

1. Take the specimen from the second pure xylene bath, gently dry its surface from the excess xylene with gauzes and place it in the first melted Paraffinmix bath taken from the laboratory oven. Place the container back in the oven. Leave it for 11 days at 54 °C.

EXCLI Journal 2024;23:612-623 – ISSN 1611-2156
Received: September 20, 2023, accepted: April 18, 2024, published: April 29, 2024

2. Outside the oven, prepared in advance, take the specimen from the first melted Paraffinmix bath and place it in the second melted Paraffinmix bath. Place containers back in the oven. Leave the specimen there for 10 days at 54 °C.

3. Outside the oven, prepared in advance, take the specimen from the second melted Paraffinmix bath and place it in the melted Paraffinwaxmix bath. Place containers back in the oven. In addition, the Paraffinwaxmix mold is placed under the glass dome; using manometer with valve apply -45000 Pa pressure for 11 days at 58 °C.

V. Solidification

1. Carefully normalize pressure. Put the glass dome aside. Take the Paraffinwaxmix mold and place it in refrigerator for 6 hours at 4-6 °C.

2. Take the mold from the refrigerator, release the solidified block from the mold, and store the block for at least two weeks at ambient temperature.

3. Drill four holes perpendicular to the blockface in the corners of the paraffin block. Fill them with black-colored paraffin using the syringe with G18 needle.

Tissue processing schedule

	Dehydration						Clearing			Paraffin Infiltration		
Reagent	Ethanol (%)						xylene 50 %, ethanol 50 %	xylene		42-44MP 50 %, 57-58MP 50 %		42-44MP 60 %, 57-58MP 25 %, Beeswax 15 %
	70	80	90	96	abs.	abs.						
Duration (days)	3	4	3	4	3	4	3	4	3	11	10	11
Agitation	yes				no		no			no		
Depression	no				-45000 Pa		no			no		-45000 Pa
Temperature	Ambient (22-24 °C)						Ambient (22-24 °C)			54 °C		58 °C

PARAFFIN RECIPES

Paraffinmix
This mix is used for first two bathes of paraffin immersion.

Paraffinmix approx. 50 °C MP – 1 L
=
Paraffin 42-44 °C MP Merck® – 0.5 L
+
Paraffin 57-58 °C MP Histoplast® – 0.5 L

Paraffinwaxmix
This mix is used for last bath and vacuuming to create paraffin block.

EXCLI Journal 2024;23:612-623 – ISSN 1611-2156
Received: September 20, 2023, accepted: April 18, 2024, published: April 29, 2024

Paraffinwaxmix 50 °C MP – 1 L
=
Paraffin 42-44 °C MP Merck® – 0.6 L
+
Paraffin 57-58 °C MP Histoplast® – 0.25 L
+
Beeswax white 61-65 °C MP WVR® – 0.15 L

SECTION CUTTING AND PHOTOGRAPHING

Important!

- Only trained personnel is allowed to operate the microtome.
- Microtome knife is handled only while wearing cut resistant gloves.
- All the manipulations with the microtome (outside of cutting procedure) should be done only when the microtome is in a Manual mode (see microtome manual).

Useful information:

Manuals for Canon 80D camera, EOS Utility 3 software, and Leica SM2500 microtome can be found in laboratory repository or on the manufacturers' websites.

I. The set-up

1. Install the specimen on the microtome and align it to be in a horizontal plane (see microtome manual for instructions).
2. Check the charge of all batteries used in camera's peripherals.
3. Install the camera on the stand and connect it to the laptop via USB port, attach flash-master module, install flashes and connect them to external power adapters.
4. Switch on the microtome, install microtome knife, adjust it and trim the block (see microtome manual).
5. Switch on only the main light in the room and close window shutters.
6. Choose Manual mode on the camera, then switch it on alongside with flashes and flash-master.
7. Launch EOS utility 3 software, click on "Remote shooting" button and set the following parameters in EOS interface:
    - Aperture – 5.0,
    - ISO – 100,
    - Shutter speed – 1/60,
    - White balance – K5500,
    - Picture style – Fine detail,
    - HDR Mode – Disabled,
    - WB SHIFT – "0,0",

- Image quality – L (6000 x 4000 px), file extension - .jpg
- Metering mode – Evaluative,
- Drive mode – Single shooting.

8. Then click on "Live View shooting" button, and a new window will be opened. Choose the Aspect ratio 3:2, and again check White balance settings. If needed set Color temperature to K5500.
9. Align camera field-of-view with the paraffin blockface.

II. Obtaining blockface images

1. Cut the paraffin block (see microtome manual): 10 iterations of 20 micron cuts (200 microns).
2. Make the camera focus on the blockface surface. For this move the AF (auto-focus) point marker on the area with good contrast (sulci, the nucleus striatus etc.), choose FlexiZone – Single in Focus parameters and click on "ON" button. *Important!* Do not focus on the white matter with few vessels or on paraffin: this will result in blurred photographs.
3. Switch off main light in the room.Click on shutter-release button to shoot the photo. Switch on main light in the room.
4. Reset the microtome (see microtome manual).
5. Repeat the steps 1-4 until the whole paraffin block is cut or the session ends.

III. Finishing

1. Switch off the camera, flashes and flash-master.
2. Take away all the batteries, disconnect external power adapters.
3. Dismantle microtome knife and clean it with 96 % ethanol, then carefully apply liquid paraffin oil to the cutting edge. Indicate on the box for knives which one was used and usage date.
4. Switch off the microtome and clean it.
